# Known-Group Validity and Sensitivity to Change in the Sensory-Motor Dysfunction Questionnaire in Individuals with Neck Pain: A Pilot Study

**DOI:** 10.3390/brainsci14111050

**Published:** 2024-10-23

**Authors:** Ushani Ambalavanar, Megan McIntosh, Heidi Haavik, Bernadette Murphy

**Affiliations:** 1 Faculty of Health Sciences, Ontario Tech University, Oshawa, ON L1G 0C5, Canada; 2 Center of Chiropractic Research, New Zealand College of Chiropractic, Mount Wellington, Auckland 1060, New Zealand

**Keywords:** spine pain, sensorimotor integration, questionnaire, known-group validity, sensitivity to change

## Abstract

Background/Objectives: Sensorimotor dysfunction is often reported in individuals experiencing neck pain, irrespective of severity and chronicity. The treatment of neck dysfunction has been found to positively impact sensorimotor integration (SMI), thereby improving sensorimotor function. However, no patient-reported outcome measure has been validated for discrimination between healthy individuals and neck pain patients with disordered SMI, nor has there been documentation of positive change in SMI following neck pain treatment.The purpose of this study was to administer the Sensory-Motor Dysfunction Questionnaire (SMD-Q), for the purposes of: (1) known-group validity between individuals without and with chronic neck pain (CNP) or subclinical neck pain (SCNP); (2) assess the questionnaire’s capacity to quantify changes in altered SMI following a tailored treatment plan. Methods: Part 1—Known Group Validity: The SMD-Q was administered to 30 neck pain (13 with CNP, and 17 with SCNP), and 30 healthy participants. Part 2—Sensitivity to Change: The SMD-Q was re-administered to neck pain participants following their tailored treatment plans (SCNP—8-weeks and CNP—12 weeks). Results: The SMD-Q can discriminate between healthy and neck pain participants (*p* ≤ 0.001), and may be sensitive to showing treatment effects (η_p_^2^ = 0.162; large effect size (ES)), but the sample size was too small to determine if it can discriminate treatment effects between groups (η_p_^2^ = 0.070; medium ES). Conclusions: Differing degrees of disordered SMI can be discriminated by the SMD-Q, but further research is needed to determine its sensitivity to treatment.

## 1. Introduction

Neck pain is a great personal burden and the fourth highest cause of disability [1]. Neck pain may arise from the continual stress from forward bending and/or abnormal positioning for prolonged periods [2,3,4,5], resulting in chronic neck pain via maladaptive motor control strategies/kinematics, and/or plasticity, if left untreated [6,7]. The persistence, worsening, or recurrence of neck pain may be due to processing of altered proprioceptive input within the central nervous system for sensorimotor integration, multimodal integration, and motor control [8,9,10].

Sensorimotor integration (SMI) is a complex process in which the central nervous system (CNS) produces task-specific motor outputs based on the quick and selective combination of sensory data from various sensory inputs [11]. SMI uses both feedforward and feedback control [11] to filter sensory inputs based on their intended function and destination. Feedforward control establishes a movement plan in advance (based on past experience and/or information), whereas feedback control relies on active sensory feedback while the action is being executed (for movement correction) requiring an accurate internal model for precision movements [12]. Through the continuous integration of afferent information, the CNS modulates the internal model (body schema) to update motor responses [11]. Multi-modal integration (MMI) is a subprocess of SMI and refers to the integration of information from various sensory modalities altering the perception of, and response to, those stimuli [13,14,15].

Disordered SMI from recurrent neck pain has been documented as deficits in central and cortical SMI [16,17,18,19], MMI [20], motor control [21], limb SMI and motor performance [22,23,24], and sensorimotor function [25,26,27,28,29,30,31,32,33]. The same has also been observed in those with chronic neck pain [34,35,36], which is possibly related to the altered neck sensorimotor control [37] from the reorganization of neural networks [38]. Altered sensory input from the neck, and deep paraspinal tissues within the vertebral column, are proposed to initiate maladaptive changes [10,39], which are seen as greater sensorimotor disturbances in those with chronic pain versus acute or recurrent pain.

The treatment of neck pain via spinal manipulation of dysfunctional vertebral segments impacts the neurophysiological response of the central nervous system [40,41], eliciting improvements in the sensorimotor processing [42,43] and function [39,44], including upper limb proprioceptive awareness [45] and motor control [46]. Spinal manipulation is applicable for milder forms of spinal dysfunction but is not applicable for more serious vertebral lesions such as myelopathies and inflammatory arthritis [47,48,49] that may require neurorestorative therapies [49]. Manipulation stimulates Golgi tendon organ afferents, and muscle spindle afferents, while smaller-diameter sensory nerve fibers are also likely to be engaged [40]. It also decreases pain perception and reverses deficits in SMI to improve motor control/function, proprioception, and the mobility of joints [40,50]. Past work has found that neurophysiological changes begin at four weeks of treatment with more substantial and ongoing improvements at week 12 [28,51,52]. Neurophysiological changes include the normalized central processing of somatosensory information [52], increased MMI [28,53], improved sensorimotor function (e.g., mobility and performance of acquired motor tasks) [28,51,53], and increased kinesthetic awareness of the lower limbs [28]. Many studies have shown immediate changes following a single treatment session involving the spinal manipulation of dysfunctional joints [19,42,43,46]. These changes seem to reflect an immediate improvement in accuracy of perception [39,40], such as improvement in elbow joint position sense [45]. With other more complex changes, such as the accuracy of perceiving sound and visual information simultaneously, changes occurred after four weeks of treatment [28]. However, with even more complex tasks (such as the ability to take a rapid step in response to a light), significant improvement had not occurred after four weeks, yet there was substantial improvement after 12 weeks of treatment [28]. These studies suggest that some improvements occur rapidly, while for more complex neurophysiological processes, improvements require repeated treatments over longer time periods to reverse maladaptive neuroplastic changes.

These differences in sensorimotor function in those with varying types of pain, as well as neurophysiological improvements in response to spinal manipulation, have been measured objectively in various laboratory settings. Until recently, there was no patient-reported outcome measure (PROM) that documented, quantified and/or assessed the disordered SMI and MMI that may accompany recurrent or chronic spinal problems. The Sensory-Motor Dysfunction Questionnaire (SMD-Q) filled this gap [54], and it was found to reliably capture disordered SMI and MMI in subclinical neck pain (SCNP) participants [54].

It is unknown whether the SMD-Q is able to discriminate between those with, and without, disordered SMI, specifically those with SCNP or chronic neck pain (CNP). The purpose of the first part of this study was to assess the known-group validity of this questionnaire in those with and without recurrent neck pain or CNP. It was hypothesized that the questionnaire would be able to discriminate healthy participants from those who have neck pain (whether recurrent or chronic) and between neck pain subgroups.

Building on this, the second part sought to determine whether the SMD-Q is sensitive to change or responsive with respect to treatment, 8 weeks for SCNP and 12 weeks for CNP groups. It was hypothesized that the SMD-Q would be able to capture the normalization of disordered SMI following treatment and document differing magnitudes of treatment effect between patient groups.

## 2. Materials and Methods

### 2.1. Participants

SCNP individuals were closely involved with the original development of this questionnaire in both piloting and focus group interviews [54]. SCNP refers to repeated episodes of mild-to-moderate neck pain that persist for at least 3 months for which individuals do not seek treatment [55,56]. CNP refers to persistent episodes of severe neck pain that persist for more than 3 months [57,58]. Individuals with SCNP and CNP served as research participants for the first and second parts of the current study.

CNP participants were recruited from 14 private practices (focused on chiropractic and/or physical therapy) in the Greater Toronto Area. Recruitment posters were distributed to the private practices via email to be posted within their clinics. Participants who were between the ages of 18 and 65, diagnosed with CNP (severe neck pain greater than 3 months), and not sought treatment in the past month were eligible to participate. Participants were recruited between 1 December 2021 and 2 May 2022. Interested participants scanned the QR code on the recruitment poster using their cellular device via a mobile browser, where they were able to read the consent form before completing the pre-screening questionnaire and were prompted with the chronic pain grade scale and neck disability index (used to confirm eligibility), which was followed by the SMD-Q. This study was approved by the Ontario Tech University Research Ethics Board (REB File #: 16575).

The healthy and SCNP participants’ datasets were acquired from another study [59], which was conducted between 7 September 2021 and 30 March 2022. Participants from that study provided consent for secondary data use (REB #:14686). Individuals between the ages of 18 and 35, who did or did not experience recurrent/ongoing periods of neck pain and/or stiffness for at least 3 months or more and have not sought treatment for this dysfunction, were eligible to participate in that study, and their SMD-Q data were included in this study.

The Von Korff Chronic Pain Grade Scale (CPGS), a 7-item self-report questionnaire, was used to quantify the severity of neck pain and pain-related disability over the span of 6 months [60]. Individuals who classified as grade 0 (pain-free), grade I (low disability–low intensity), grade II (low disability–high intensity), grade III (high disability–moderately limiting) or grade IV (high disability-severely limiting) were eligible to participate. The Neck Disability Index (NDI), a 10-item self-report questionnaire, was used to determine neck-pain related disability at the moment of administration [61,62] and discriminate between SCNP and CNP at baseline. Individuals who scored between 5 and 24 confirmed that SCNP participants exhibit mild-moderate disability from their neck pain episodes, while a score greater than 25 confirmed CNP participants exhibit severe disability from their severe neck pain [61]. None of the participants were to have any accompanying neurological conditions that are known to impact cognitive function and/or central somatosensory processing (e.g., multiple sclerosis, stroke, residual symptoms from a head injury, etc.) at baseline and post-treatment (for those with dysfunction following their treatment plan).

CNP participants provided informed consent electronically by clicking “I agree” upon reading the electronic consent form before starting the series of questions and questionnaires.

### 2.2. Experimental Protocol

SCNP, CNP and healthy participants completed the SMD-Q using Qualtrics™ software (Qualtrics, Provo, UT, USA. https://www.qualtrics.com/), version September 2021–May 2022 at baseline. SCNP and CNP participants proceeded to receive treatment for their spinal dysfunction, which spanned over 8 weeks for SCNP and 12 weeks for CNP. Upon completion of their treatment, the SMD-Q was re-administered (sent via email) at post-treatment.

#### 2.2.1. Treatment for Patients with Neck Dysfunction

CNP participants received 12 weeks of treatment from a registered practitioner and adhered to the practitioner’s recommended treatment plan. Treatment plans were tailored by their treating clinician to address their presenting concerns accordingly. Participants’ reported receiving chiropractic care, massage therapy, and physiotherapy.

SCNP participants from the other study (REB #: 14686) underwent chiropractic care for 8 weeks. Their treatment plan comprised adjustments to dysfunctional segments in the spine as well as myofascial release of the associated muscles, as clinically indicated. Participants reported only receiving chiropractic care.

#### 2.2.2. The Sensory-Motor Dysfunction Questionnaire (SMD-Q)

The SMD-Q was created to be administered to individuals with chronic or recurrent spine pain to determine the degree to which a person’s altered spinal function may be affecting his/her central processing and in turn affecting their daily life functioning [54].

The SMD-Q questionnaire comprised 12 items surrounding constructs of SMI. The questions were presented as how often an item happened to them “over the past week”. Four options were provided, which included (1) Never/Rarely (<1 day); (2) Some or little of time (1–2 days of the week); (3) Often or a moderate amount of time (3–4 days of the week), and (4) Most or all of the time (5 or more days of the week); see Appendix A. The item responses were transformed to a score from 0 to 3. The total score was calculated by summing the score of each item. The maximum score is 36. A higher score is indicative of a greater degree of dysfunction with respect to SMI.

### 2.3. Statistical Analysis

There were no missing items for any of the participants who completed the SMD-Q at baseline and post-intervention. All statistical analyses were performed with IBM SPSS Statistics 24.0 [63]. Statistical significance was set at *p* ≤ 0.05.

#### 2.3.1. Known Group Validity

Descriptive statistics and Kolmogorov Smirnov test of normality (*n* > 50) was used to examine the distribution of the continuous data, revealing a non-normal distribution. The Independent-Samples Kruskal-Wallis H Test was used to determine whether the total scores of the three groups (CNP, SCNP, and healthy) were related to one another, and perform pairwise comparisons (Healthy & SCNP, Healthy & CNP, and SCNP & CNP). SPSS adjusted the *p*-value by the Bonferroni correction to correct for multiple independent comparisons [63].

#### 2.3.2. Sensitivity to Change

Descriptive statistics and the Shapiro–Wilk test were used to examine the distribution of the continuous data (total scores of SMD-Q) at baseline and follow-up. The follow-up data was non-normally distributed. Both datasets were transformed using a square root transformation before statistical testing was performed. A 2 (group) × 2 (time) repeated measures analysis of variance (ANOVA) with a pre-planned contrast to baseline was conducted to examine the effect of treatment for all neck pain participants and between the two neck pain groups (CNP and SCNP) with varying treatment durations. Partial eta squared (η_p_^2^) was reported, reflective of effect size, where 0.01 is small, 0.06 is medium, and 0.14 is large [64].

The standardized response mean (SRM), an effect size index, was used to gauge the responsiveness of the SMD-Q [65]. It is calculated by dividing the change in mean (*µ*) by the change in standard deviation (ϑ). A negative SRM value was considered a decrease in dysfunction, while a positive SRM value is indicative of an increase in dysfunction. SRM was performed in Microsoft Excel, and the formula is as follows:

(1) $$\mathrm{SRM}=\frac{\mu 2-\mu 1}{\vartheta 2-\vartheta 1}$$

Note: The numbers beside the symbols indicate the time point, 2 refers to the post-treatment time point, and 1 refers to the measure at baseline.

## 3. Results

Descriptive results are written as mean ± standard deviation.

### 3.1. Part 1: Known-Group Validity

#### 3.1.1. Demographic and Neck Pain Characteristics

All 60 participants (33 F and 27 M; age: 25.9 ± 9.3 years of age) completed the questionnaire at baseline and were included in the analysis. See Table 1 for the demographics of each group.

#### 3.1.2. SMD-Q: Total Score

**Figure 1:**
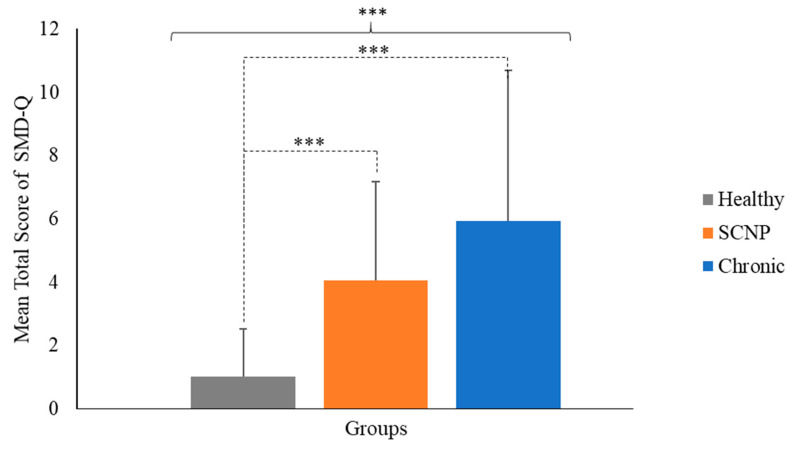
Mean score of the SMD-Q, where gray is the healthy group, orange is the SCNP group, and blue is the CNP group. The error bar represents SD. Asterisks and square bracket indicate the three groups are significantly different from one another. Asterisks and dashed brackets indicate a significant difference between two groups. *** *p* < 0.001.

The Kruskal-Wallis H test showed that there was a statistically significant difference in total scores of the SMD-Q between the groups [χ^2^(2) = 20.70, *p* < 0.001], with a mean score of 5.92 ± 4.77 for the CNP group, 4.06 ± 3.13 for the SCNP group, and 1.03 ± 1.50 for the healthy group, see Figure 1. The Kruskal–Wallis H test also indicated a statistically significant difference in the total score of this questionnaire between healthy and SCNP participants as well as healthy and CNP participants; see Figure 1 and Table 2.

### 3.2. Part 2: Sensitivity to Change

A total of 22 individuals (11 F and 11 M; age: 24 ± 5.5 years) with neck pain completed the questionnaire at baseline and following treatment (8 or 12 weeks later). Out of the 22 participants, 8 individuals (4 F and 4 M; age: 29.12 ± 6.12 years) had CNP and 14 individuals (7 F and 7 M; age: 21.43 ± 2.14 years) had SCNP. The duration/onset of pain for the CNP and SCNP was 3.59 ± 4.41 years and 1.73 ± 1.62 years, respectively.

#### SMD-Q: Total Score

**Figure 2:**
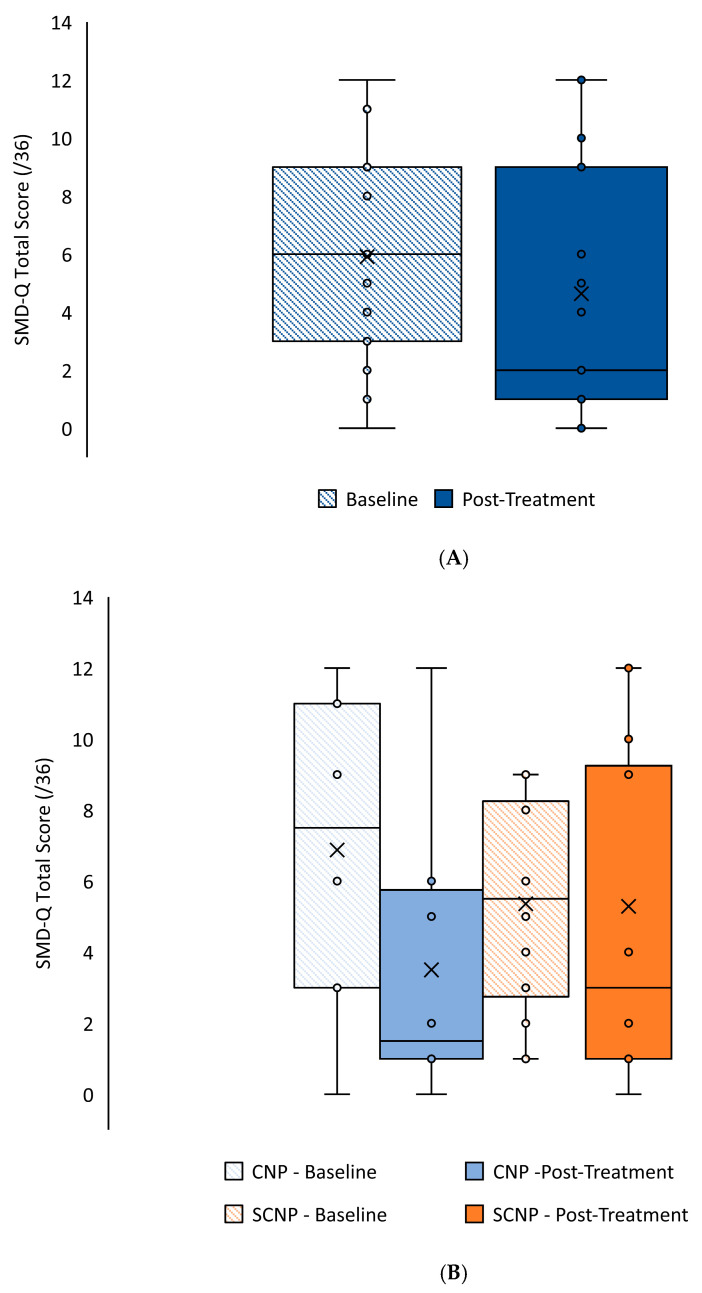
Distribution of the SMD-Q total score, (**A**) per time for the sample, and (**B**) per group and time. The whiskers reflect minimum and maximum values. The circles denote the inner points. The boxes reflect the quartiles, and the × symbol represents the mean.

The repeated measures ANOVA demonstrated a large effect size and a near-significant effect of time (F _(1, 20)_ = 3.860, *p* = 0.06, η_p_^2^ = 0.162) with a 21.54% reduction in scores between baseline (5.91 ± 3.57) and post-treatment (4.64 ± 4.37); see Figure 2A. There was a medium effect size but no significant time by group interaction (F _(1, 20)_ = 1.507, *p* = 0.234, η_p_^2^ = 0.070); see Figure 2B. The SCNP group had a 1.33% score reduction post-treatment, while the CNP group had a 49.09% reduction (see Table 3 for mean scores).

The mean difference for all neck pain participants combined (CNP and SCNP) was −1.27, and the 95% confidence interval was [4.42, 7.40] at baseline and [2.81, 6.47] at post-treatment. The total score of the neck pain participants was responsive to treatment, which was seen as an SRM value of −1.59 (reduction in degree of SMI dysfunction).

## 4. Discussion

This study demonstrated that the SMD-Q can distinguish individuals with and without neck pain (SCNP or CNP). The known group validity coefficients demonstrated that the total scores of the SMD-Q of each group were unrelated, revealing differences between individuals with neck dysfunction and healthy individuals. The total score of the SMD-Q, which sought to quantify symptoms of altered MMI and SMI-related constructs, were impacted by 8 to 12 weeks of treatment, which was reflected by the large effect size with respect to time, medium effect size for a time by group interaction, and a large SRM value. The SMD-Q could potentially capture changes in disordered SMI in response to treatment. With the low participant numbers in the current study, it cannot be stated with certainty if it is able to discriminate between individuals with CNP vs. SCNP with differing treatment durations. This research suggests that the SMD-Q can be used to understand the link between altered sensorimotor function and spinal problems.

### 4.1. Known-Group Validity

The SMD-Q can differentiate between healthy and both SCNP and CNP. This corresponds with the various studies that have employed neurophysiological techniques (objective measures) to evaluate differences in SMI and MMI in those with and without neck pain [19,66]. A greater divide in scores was expected between the SCNP and CNP group since there is literature suggesting that there are greater impairments in SMI with increasing severity of neck dysfunction due to the greater period of maladaptive plasticity alongside persistent perception of nociceptive pain; however, this was not observed. The lack of a statistically significant difference in the SCNP and CNP pairwise comparison could be due to the small sample size, as a sample of at least 30 to 50 per group is needed when assessing for validity [67]. 

### 4.2. Sensitivity to Change

The SMD-Q total score for the combined CNP and SCNP groups decreased following treatment, which is consistent with the findings of positive changes in SMI with respect to chiropractic treatment [19,43,45,68], physiotherapy or massage therapy [51,69,70]. This questionnaire appears to capture the recalibration of the body schema in response to treatment, which is adjusted within days or weeks [71]. This is reflected by a large effect size with respect to time and large SRM value suggesting that the SMD-Q may be sensitive to quantify changes in SMI previously measured in these laboratory-based studies.

The large decrease in SMD-Q scores within the CNP group is consistent with past studies that demonstrated improvements in sensorimotor function and central processing following 12 weeks of treatment [28,52]. In contrast, the miniscule decrease in score with the SCNP group does not coincide with past work suggesting that 4 to 6 weeks is sufficient to induce changes in sensorimotor function [51,53]. Interestingly, the SCNP group had a greater data dispersion post-treatment compared to the CNP group, which might be the result of the variability in the presentation of symptoms in a subclinical population in terms of chronicity and intensity (e.g., recurrent problems for just 3 months versus for several months to years without transitioning to CNP). It is possible that the SCNP participants whose onset of pain was longer than 3 months may require a longer treatment period (e.g., 12 weeks) to reverse the maladaptive presentations from disordered sensorimotor processing of altered afferent input from the musculature, which is similar to the findings of Holt et al. [28]. The medium effect size of the time by group interaction could also be attributed to a type II error due to the small sample size in each subgroup. Based on the effect size acquired from this statistical analysis, a power analysis for a repeated measures within-between interaction (2 groups and 2 measurements) with an f-effect size of 0.27 (calculated from η_p_^2^ = 0.070), and power set at 0.95 (high, to minimize type II error) indicates that 23 participants per group are needed to determine if the SMD-Q can discriminate treatment effects between CNP and SCNP.

The authors advise that a more homogenous sample of SCNP participants (i.e., presenting with similar symptoms and/or similar pain duration) be utilized in future research. In addition to this, a larger sample size is needed to confirm these findings, i.e., a minimum of 23 per group.

### 4.3. Limitations

One of the limitations of these studies is the small sample sizes. The known-group validity study did not have a sample ≥ 30 for the two neck pain subgroups. In contrast, the sensitivity to change study comprised a heterogeneous sample as opposed to two subgroups, and power calculations indicated that at least 23 participants per group would be needed. Due to this, the findings from this study cannot confirm whether the total score of the SMD-Q is able to differentiate between CNP and SCNP nor whether there are differences in the level of impact treatment has on SCNP versus CNP.

The way in which the questions are posed could account for the lack of group differences between the neck pain populations. Future research could also consider posing the question as “in a typical/usual week” alongside “in the past week” to more accurately capture the variability in sensorimotor symptoms that are experienced in an SCNP population. This is worth considering due to the recurrent nature of SCNP, where they may have minimal sensorimotor disturbances in the previous week, despite experiencing it frequently when thinking in the context of a typical or usual week.

The administration of the NDI at the second time point (post-intervention) would have been helpful, enabling us to capture changes in neck pain in response to the intervention, at the moment of administration. The NDI was originally used as an inclusion criterion, as the primary focus of this study was on the SMD-Q, which is why it was not administered again post-treatment. Future studies should include a measure of neck pain and disability along with the SMD-Q.

Additional clinical characteristics of the neck pain populations should also have been captured, to gain more insight in the diverse symptom presentation between the groups. As the primary focus of this work was to examine the known-group validity and assess the responsiveness of the SMD-Q, and we did not have access to the patient’s clinical records, this information was not able to be collected.

The CNP participants did not provide detailed information regarding their treatment plans; rather, they stated that they received various types of treatment including chiropractic treatment, physiotherapy, and massage therapy. Future studies could consider controlling the type, duration and/or frequency of treatment received as this may provide greater insight on the degree of SMI improvement with respect to specific treatment–dose relationships.

## 5. Conclusions

The findings of this study indicate that SMD-Q can differentiate between healthy participants and those with varying types of neck pain, e.g., SCNP and CNP. This questionnaire may be sensitive to changes following a tailored treatment plan in those with either SCNP or CNP but not differences in the level of impact treatment has on SCNP versus CNP. However, this cannot be stated with certainty due to the large variability in symptoms within the SCNP group, the differing treatment durations, and the differing sample size between the groups. The work does suggest that the SMD- Q may be a useful clinical measure of altered SMI and MMI and a potential new clinician administered tool to be used in clinical settings alongside existing interventions and/or assessments. Subsequent studies should implement the proposed change and include a larger sample size (e.g., a minimum of 23 participants per group and/or subgroup).

## Figures and Tables

**Table 1:** Descriptive of sample.

			Groups		
			Healthy	SCNP	CNP
Number of participants			30	17	13
Biological Sex Ration (F:M)			15:15	10:7	7:6
Age (y; mean ± SD)			21.33 ± 2.55	21.23 ± 1.97	31.77 ± 8.94
Duration/Onset of Pain (months)			0.00 ± 0.00	21.63 ± 18.14	71.69 ± 94.53
NDI Score (/50)			0.70 ± 1.13	6.59 ± 4.32	29.15 ± 8.92
Von Korff Chronic Pain Grade Scale					
	Characteristic Pain Intensity		2.10 ± 5.04	51.40 ± 13.73	59.23 ± 17.24
	Disability Score		0.00 ± 0.00	0.75 ± 0.97	1.21 ± 0.93
	Disability Points		0.00 ± 0.00	1.33 ± 0.89	0.77 ± 0.93
	Pain Grade Classification				
		Grade 0	30	0	0
		Grade I	0	11	3
		Grade II	0	5	10
		Grade III	0	1 *	0
		Grade IV	0	0	0

* This participant experiences bad pain episodes reflected by a high pain grade classification; however, the SMD-Q was administered on a pain-free day where they were asymptomatic.

**Table 2:** Pairwise comparisons.

	Test Statistic	*p*	Mean Difference	Confidence Intervals
Healthy-SCNP	18.29	<0.001	3.03	[1.67, −4.39]
Healthy-CNP	21.93	<0.001	4.89	[2.96, −6.82]
SCNP-CNP	3.65	0.562	1.86	[−1.10, −4.82]

**Table 3:** SMD-Q Scores per group, and time point.

	Baseline	Post-Treatment	Difference Scores
SCNP	5.36 ± 2.95	5.29 ± 4.56	−0.07 ± 3.97
CNP	6.88 ± 4.52	3.50 ± 4.04	−3.38 ± 5.29

## Data Availability

The data can be made available by the corresponding author upon reasonable request. The data are not publicly available due to ethical and privacy considerations.

## Appendix A

The Sensory-Motor Dysfunction Questionnaire (e.g., Questions About your Brain–Body Communication)

This questionnaire seeks to determine to what degree your spinal dysfunction may be affecting how you process sensory information and therefore how it is affecting your function. Instructions: Please answer ALL the scales, by choosing the option that is most applicable to you, in the past week.

	Never/Rarely When Doing This Task/Action (<1 Day)	Some or Little of the Time When Doing This Action/Task (1–2 Days)	Often or a Moderate Amount of Time When Doing This Action/Task (3–4 Days of the Week)	Most or All the Time When Doing This Action/Task (5 or More Days of the Week)
Question 1: Over the past week, on average, how often did you have problems with your physical balance (i.e., frequent loss of balance or unsteadiness or feel like you might fall while walking, running or standing still, etc.)?	□	□	□	□
Question 2: Over the past week, on average, how often did you have hand-eye coordination problems (e.g., several typos when typing on your phone or computer keyboard, several mistakes when playing an instrument while reading sheet music, reading and miswriting, mis put key into door lock to unlock the door, making small mistakes when playing a sport with the hands, etc.)?	□	□	□	□
Question 3: Over the past week, how often have you accidentally bumped your head into things (i.e., hitting the top of your head when getting out of the car, bumping your head into a kitchen cupboard, etc.)?	□	□	□	□
Question 4: Over the past week, how often have you bumped into people or objects/things (i.e., table, wall, chair or leg of a chair, etc.) with other parts of your body?	□	□	□	□
Question 5: Over the past week, how often have you missed when you reached for an object without looking (e.g., phone, water bottle, cup, pen, book, kitchen tools, keys, etc.)?	□	□	□	□
Question 6: Over the past week, how often did you fail to pick something up that you initially dropped (i.e., slipped out of your hands or butter fingers, etc.)?	□	□	□	□
Question 7: Over the past week, how often have you missed when you had to use your leg or foot (i.e., misplaced or awkward step when walking or walking up/down the staircase or stepping up to a chair or stool, missed when kicking a ball or putting your foot in slip-on shoes, pushing against the gas pedal instead of the brake pedal, etc.)?	□	□	□	□
Question 8: Over the past week, how often have struggled to perform a movement you normally do well (e.g., missed when hitting or catching a ball, tripped while walking or running, struggled with a musical instrument you normally play well, pushing against the gas or brake pedal too hard or soft, biting the side of your mouth, lip or tongue while eating, etc.)?	□	□	□	□
Question 9: Over the past week, how often have you had trouble recognizing familiar sounds that you normally recognize very quickly (e.g., mishearing words or phrases when someone is speaking to you or mishearing lyrics of a familiar song)?	□	□	□	□
Question 10: Over the past week, how often have you had trouble recognizing familiar objects, places or people that you normally recognize very quickly (e.g., recognizing that a cat ran past as opposed to just a fast-moving object or recognizing familiar face(s) when you walk past them at the grocery store, in the hallways at work or school, or recognize the place that you frequently pass by, etc.)?	□	□	□	□
Question 11: Over the past week, how often have you been having difficulties concentrating in situations where there is a lot of background noise (e.g., people coughing, side conversations around you, driving while listening to music and passengers having a conversation with you, etc.)?	□	□	□	□
Question 12: Over the past week, how often have you had difficulties performing tasks that require you to combine information from more than one sense (sound, sight, smell, taste, etc.) at the same time (i.e., you aren’t as fast at combining sight and sound information from traffic so you have difficulty gauging how much time you have to cross the street, or during online gaming; or food is not smelling and tasting as it used to, or you are not as accurate at judging the space available to pass through an opening so you bump into things, etc.)?	□	□	□	□
